# EZH2 Might Affect Macrophage Chemotaxis and Anti-Inflammatory Factors by Regulating CCL2 in Dental Pulp Inflammation

**DOI:** 10.1155/2021/3060480

**Published:** 2021-12-01

**Authors:** Ziqi Hu, Yingyi Chen, Jie He, He Liu, Tianqian Hui

**Affiliations:** Department of Pediatric Dentistry, Central Laboratory, Peking University School and Hospital of Stomatology & National Center of Stomatology & National Clinical Research Center for Oral Diseases & National Engineering Laboratory for Digital and Material Technology of Stomatology, No. 22, Zhongguancun South Avenue, Haidian District, Beijing 100081, China

## Abstract

**Objectives:**

We aimed to evaluate the effects of Enhancer of Zeste Homolog 2 (EZH2) on regulation of macrophage migration and expression of anti-inflammatory genes in pulpitis.

**Methods:**

Dental pulp inflammation was verified by histology in rat pulpitis model induced by lipopolysaccharide (LPS). Immunohistochemistry staining was used to detect changes of the expression of EZH2 and tumor necrosis factor alpha (TNF-*α*) in dental pulp inflammation. The expression of EZH2, CCL2, and cluster of differentiation 68 (CD68: macrophage surface marker) was measured by immunofluorescence staining. The effect of EZH2 on microphage migration was assessed by cell migration assay. The expressions of anti-inflammatory cytokine interleukins (IL-4 and IL-10) and transforming growth factor-*β* (TGF-*β*) in HDPCs which were treated by EZH2 complex, CCL2 complex, and CCL2 antibody were examined by quantitative real-time polymerase chain reaction (q-PCR).

**Results:**

The expression of TNF-*α* gradually increased in dental pulp inflammation. The expression of EZH2 in dental pulp decreased in 8 hours after LPS stimulation. However, the expression of EZH2 gradually increased in dental pulp after 1 day stimulation by LPS. The results of immunofluorescence staining showed that the expressions of EZH2, CCL2, and CD68 were significantly upregulated in dental pulp inflammation of rats. EZH2 could enhance macrophage migration. And the chemotactic activity of macrophages exposed to supernatants of EZH2-treated HDPCs could be inhibited by CCL2 inhibition. In addition, EZH2 suppressed the expression of anti-inflammatory genes, but CCL2 inhibition reversed the downregulation of anti-inflammatory factors, including IL-4 and TGF-*β* in HDPCs.

**Conclusions:**

EZH2 might affect chemotaxis of macrophages and the expression of anti-inflammatory factors by regulating CCL2. EZH2 plays an important role in the development of dental pulp inflammation, and it might be as a target for treatment of pulpitis.

## 1. Introduction

Pulpitis is a multifactorial disease that could be mainly caused by dental caries, as well as mechanical and chemical irritations. These events, such as dental caries, can irritate dental pulp healing process if the infection is not too severe [1]. The mechanisms regulating pulpitis and repair were complicated. Dental pulp inflammation usually could persist in the dental pulp despite treatment, reducing innate repair capacities [2].

Studies have found that epigenetic regulation plays an important role in the progress of dental pulp inflammation [3]. Epigenetics is defined as a heritable change in gene function without a change in the DNA sequence, which ultimately leads to a change in phenotype [4]. Epigenetics includes DNA methylation, histone modification, and noncoding RNA. The role of histone modification in inflammation and repair has gradually attracted attention [5–7]. Histone H3 on lysine residue 27 (H3K27me) is a common site for histone modification. Some studies have confirmed that demethylation of H3K27me3 can promote the repair reaction of dental pulp. And Enhancer of Zeste Homolog (EZH2) is a trimethylation transferase of H3K27. It is the catalytic subunit of polycomb repressive complex 2 (PRC2). And EZH2 has been shown to play an important role in a variety of inflammatory diseases, such as nervous system inflammation and enteritis [8–10]. However, the mechanism of EZH2 in pulpitis is still unclear. Previous studies have shown that EZH2 promoted the progress of dental pulp inflammation [6]. EZH2 could promote the proliferation of human dental pulp cells and inhibit osteogenic differentiation [6]. In addition, EZH2 can directly combine with the promoters of IL-6, IL-8, and CCL2 to regulate the histone modification and increase expressions of the genes. Among these changing inflammatory factors, the expression of CCL2 changed most [3].

CCL2 is a chemokine of mononuclear macrophage. CCL2 could promote the chemotaxis of a large number of macrophages to accumulate at the site of the inflammatory area [11, 12]. Then, the chemokine-cytokine network is activated, which could result in the amplification and persistence of the inflammatory response [13, 14]. In dental pulp inflammation, HDPCs express chemokines including CCL2, which could be induced by LPS or TNF-*α* stimulation [15]. DPSCs exhibit their immunomodulatory effects on macrophage phenotype in inflammatory diseases [16]. HDPCs are the most numerous cells in the dental pulp and maintain the collagen matrix of the pulpal tissue, and a population of immune cells, such as macrophages, hold themselves ready to respond to microbial incursion [17]. Macrophages and neutrophils are important mediators of the innate inflammatory response in the dental pulp [18]. Macrophages stimulated with IL-10 and TGF-*β* could decrease the production of inflammatory cytokines, such as TNF-*α* in dental pulp [19]. We speculated that macrophages might play an important role in pulpitis and modulate the pulp regenerative environment. According to the current research, epigenetic reprogramming has been involved in macrophages activation [20]. It is speculated that the effect of EZH2 on dental pulp inflammation might include microphage chemotaxis. EZH2 could affect the production of inflammatory/chemokines, immune regulatory functions, and process of the pulpitis [3]. However, the regulatory mechanism of EZH2 in the process of dental pulp inflammation and immune response remains to be further studied.

In our study, we explored the chemotactic effects of EZH2 on macrophages and anti-inflammatory genes in dental pulp inflammation.

## 2. Materials and Methods

### 2.1. Construction of Dental Pulp Inflammatory Models in Rats

All animal manipulations were approved by the local ethical committee at Peking University (LA2018044). A well-characterized rat experimental pulp inflammation model was established as described previously [3]. Rats (6 weeks of age) were used and divided into 2 groups with three rats each. LPS from E.coli (10 g/L, Sigma, United States) for inflammation group (with different time points, including 2 hours, 8 hours, 1 day, 3 days, and 7 days). The control group did not have any treatment, and the number of experimental teeth in each group was not less than 5. LPSs were applied to the amputated pulp using Spongel (Astellas Pharma) and sealed with glass ionomer cement (GIC) (Fuji IX, Ketac Molar, and d-tech). Rats were sacrificed 2 hours, 8 hours, 1 day, 3 days, and 7 days after operation by decapitation. And the jaws were fixed promptly in 4% formaldehyde and then decalcified for 1 month and embedded in paraffin. Sections of 5 *μ*m thickness were stained with H-E and immunohistochemistry.

### 2.2. Histopathologic

Three consecutive sections per teeth were stained with hematoxylin and eosin (H&E) and selected for the morphometric analysis under the microscope (BX51 Olympus Micro Imaging System, Japan).

### 2.3. Immunohistochemical Analysis

The paraffin sections were baked at 65°C overnight. A standard immunohistochemistry kit (Zhongshanjinqiao, Beijing, China) was used for immunohistochemistry according to the manufacturer's instructions. Primary antibody, 1 : 50 dilution of EZH2 (Cell Signaling Technology, #5246, United States) and 1 : 100 dilution of TNF-*α* (Abcam, ab199013, UK), was added, and the slides were incubated at 4°C overnight. 3,3′-Diaminobenzidine kit (Zhongshanjinqiao) was used for coloration according to the manufacturer's instructions. Results were obtained using an Olympus BX51 upright microscope (Olympus Optical, Tokyo, Japan).

### 2.4. Immunofluorescence

For immunofluorescence staining, serial sections of 4 *μ*m thickness were incubated with antibody against EZH2 (1 : 200; Cell Signaling Technology, #5246), antibody against CCL2(1 : 200; Abcam, ab25124), and antibody against CD68 (1 : 200, Abcam, ab31630) overnight at 4°C. Bound primary antibodies were detected with Alexa Fluor 488 preadsorbed anti-rabbit IgG secondary antibody (1 : 200; Abcam, ab150117) and Alexa Fluor 647 preadsorbed anti-mouse IgG secondary antibody (1 : 200; Abcam, ab150083), following a 1 h incubation at room temperature. The sections were then counterstained with DAPI (Zhongshanjinqiao, Beijing, China); then, images were taken with a fluorescence microscope (BX51; Olympus).

### 2.5. Cell Culture

The study was approved by the ethics committee of the Peking University of Stomatology (PKUSSIRB-201732003). THP-1 cells were purchased EK-Bioscience were incubated in a medium containing 15 *μ*g/L 12-myristate 13-acetate (phorbol ester, phorbol 12-myristate 13-acetate, PMA, Sigma) for 48 hours to induce differentiating into macrophages [21]. Primary HDPCs were cultured as described previously [22], and passages 3–5 were used. Cells were treated with recombinant EZH2 (20 ng/ml, Abnova, Taiwan), CCL2 (20 ng/ml, Abnova, Taiwan), EZH2+CCL2, and EZH2+anti-CCL2 (100 *μ*g/L, Abcam, Cambridge, MA) for 24 hours.

### 2.6. Cell Migration Assay

The migration capacity of macrophages was measured in transwell chambers (3 *μ*m pore, Corning). HDPC monolayers were incubated with serum-free DMEM with or without EZH2 (20 ng/ml), CCL2 (10 *μ*g/L), EZH2+CCL2, and EZH2+anti-CCL2 (100 *μ*g/L) treatment for 48 hours in a 5% CO_2_ incubator at 37°C before collection of supernatants. After overnight culture in serum-free RPMI1640 medium, 200 *μ*l of macrophages was resuspended (2 × 10^6^/ml) and added to the upper chamber in serum-free RPMI 1640 medium, and 600 *μ*l of the supernatant of untreated or treated HDPCs was placed into the bottom wells. After 4 hours of incubation, nonmigrating cells on the upper surface of the membrane were removed, and the cells that migrated to the underside of the polycarbonate membrane were fixed with ethanol and stained with 1% crystal violet for 30 min. The mean of triplicate assays for each experimental condition was used for analysis.

### 2.7. Real-Time Polymerase Chain Reaction Analysis

Total RNA was isolated using the RNeasy mini kit (Qiagen, Valencia, CA). Complementary DNA was synthesized from RNA by using the PrimeScript RT Reagent Kit (Takara, Dalian, China). The produced complementary DNA was prepared as templates for the polymerase chain reaction using SYBR Premix Ex Taq (Takara) according to the manufacturer's instruction. Glyceraldehyde-3-phosphate dehydrogenase (GAPDH) was used as the control. Primer sequences and conditions for real-time polymerase chain reaction are shown in the supplemental material (available here).

### 2.8. Statistical Analyses

All data were presented as mean SD and compared by one-way analysis of variance tests were calculated for statistical analysis of differences by SPSS23.0 (SPSS Inc., Chicago, IL, USA). All the experiments were independently repeated at least in triplicate. *p* < 0.05 was considered to be statistically significant.

## 3. Results

### 3.1. Dental Pulp Inflammatory Model with LPS Stimulation in the Rats Was Established and TNF-*α* Expression Was Detected

**Figure 1:**
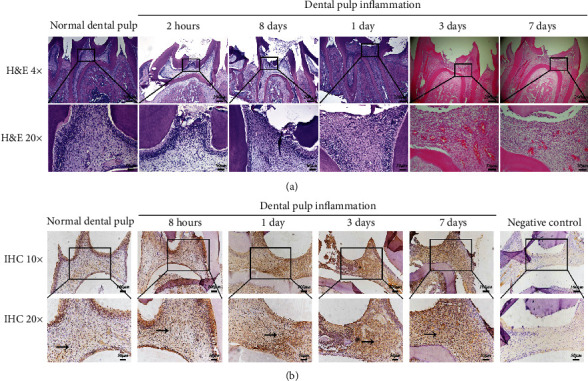
The effects of LPS on dental pulp tissue and TNF-*α* expression. (a) Hematoxylin-eosin analyses of nontreated pulp (control) and experimental rat pulpitis induced by LPS treatment observed after 2 hours, 8 hours, 1 day, 3 days, and 7 days. Higher magnifications are shown in boxed areas. Scale bar: 200 *μ*m in low magnifications and 50 *μ*m in higher magnifications. (b) Immunohistochemical analyses of TNF-*α* expression of dental pulp with or without LPS treatment after 8 hours, 1 day, 3 days, and 7 days. Scale bar: 100 *μ*m in low magnifications and 50 *μ*m in higher magnifications (*n* = 3).

To observe the effect of LPS on dental pulp tissue, histologic observations were carried out at different time points after treatment with LPS (Figure 1(a)). In LPS-treated dental pulp tissue, the areas of inflammatory cell infiltration were larger than controls. Then, the expression of TNF-*α* was detected (Figure 1(b)). The immunohistochemical staining results indicated that the expression of TNF-*α* was increased in response to LPS treatment. These results showed success of construction of dental pulp inflammatory models in rats.

### 3.2. Changes of EZH2 Expression in Dental Pulp of Rats with LPS Treatment

**Figure 2:**
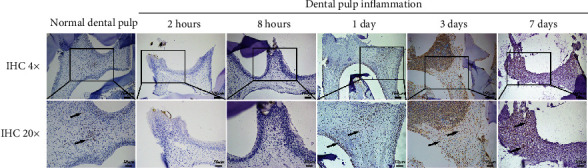
Immunohistochemical staining showed that EZH2 expression was significantly increased after 1 day stimulation. LPS stimulated rat dental pulp at different time points (2 hours, 8 hours, 1 day, 3 days, and 7 days). Black arrows indicate positive EZH2 staining. Higher magnifications are shown in boxed areas. Scale bar: 100 *μ*m in low magnifications and 50 *μ*m in higher magnifications (*n* = 3).

We investigated the involvement of EZH2 in dental pulp inflammation using immunohistochemistry staining. The EZH2 protein was lowly expressed in the nucleus of normal dental pulp but increased in inflamed dental pulp tissue (Figure 2). EZH2 has a lower expression in 8 hours compared with control. After 1 day, 3 days, and 7 days, EZH2 expression was increased obviously in inflamed dental pulp tissue. These results suggested that EZH2 might play a role in dental pulp inflammation.

### 3.3. Macrophages Are Involved in Dental Pulp Inflammation

**Figure 3:**
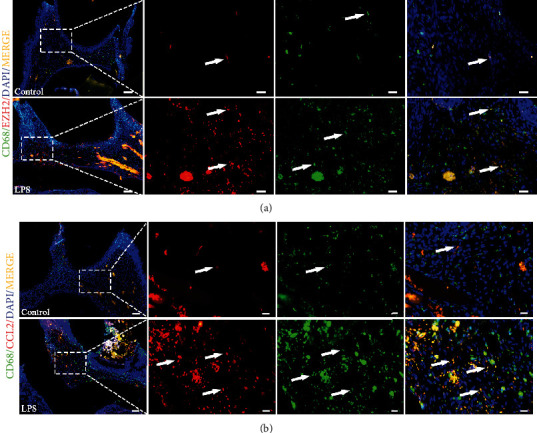
Immunofluorescence staining of rats' pulp tissues with LPS stimulation and control. Immunopositivity of (a) EZH2, (b) CCL2, and (a, b) CD68 in healthy and inflamed pulp tissues. Healthy and inflamed pulp tissues were stained with antibody to EZH2, CCL2 (red), CD68 (green), and colocalization (yellow). White arrows indicate positive staining. Higher magnifications are shown in boxed areas. Scale bar: 100 *μ*m in low magnifications and 20 *μ*m in higher magnifications (*n* = 3).

To reveal further relationship between the macrophages and pulp inflammation, CD68, a common surface marker for macrophages was detected as the target protein. Compared with the control group, the expression of EZH2 and CD68 was significantly upregulated in inflamed pulp tissue (Figure 3(a)). Then, we found the expression of CCL2 and CD68 was increased with LPS stimulation as compared with the control group (Figure 3(b)). These results indicated that the macrophages might be involved in inflamed dental pulp tissue. And EZH2 might be involved in macrophages in dental pulp.

### 3.4. EZH2-Treated HDPCs Enhanced Macrophage Migration via Regulating CCL2

**Figure 4:**
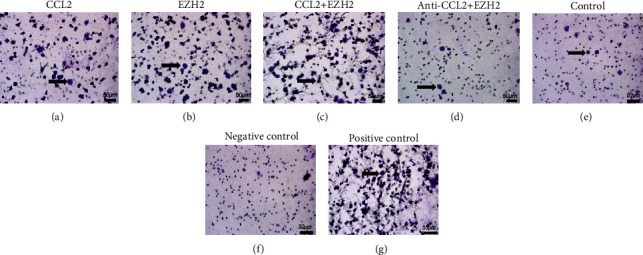
Chemotactic activity of EZH2 and CCL2-treated HDPCs on macrophages. (a) Transwell assay showing that CCL2 promotes macrophage migration. (b) Transwell assay showing macrophage migration in response to treatment with supernatants of EZH2-treated HDPCs. (c) Cell migration was induced by treatment with CCL2 plus EZH2. (d) Cell migration was suppressed by treatment with anti-CCL2 protein. (e) Cell migration of macrophages in response to HDPCs without any treatment, as control group. (f) Negative control group. (g) Positive control group. Scale bar: 50 *μ*m (*n* = 3).

We investigated the effects of EZH2 and CCL2 on macrophage migration, which has been as a hallmark of the immune response. We examined the chemotactic migration of human macrophages in response to supernatants of HDPCs that were treated with EZH2, CCL2, EZH2+CCL2, and EZH2+anti-CCL2 by a transwell migration assay. The migration of macrophages toward the supernatant of CCL2-treated HDPCs was enhanced compared with untreated HDPC supernatants (Figure 4(a)). The chemotactic activity of the supernatant of EZH2-treated HDPCs was increased when compared with control (Figure 4(b)). The chemotactic activity of the supernatant of CCL2 plus EZH2-treated HDPCs was increased when compared with control (Figure 4(c)). As shown in Figure 4, pretreatment of anti-CCL2 could inhibit the chemotaxis of macrophages that was induced by EZH2 (Figure 4(d)). These data indicate that the chemotactic activity of macrophages exposed to supernatants of EZH2-treated HDPCs could be inhibited by CCL2 inhibition.

### 3.5. EZH2 Might Affect the Expression of Anti-Inflammatory Factors in HDPCs via CCL2

**Figure 5:**
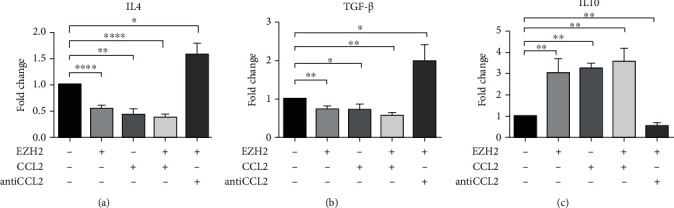
Interleukins were influenced by EZH2, CCL2, and anti-CCL2 in HDPCs. Relative mRNA expression levels of (a) IL-4, (b) TGF-*β*, and (c) IL-10 were assessed by q-PCR in HDPCs treated by EZH2, CCL2 complex, EZH2 plus CCL2 complex, and EZH2 plus anti-CCL2 for 24 hours. mRNA expression levels were normalized to GAPDH (*n* = 3).

We confirmed the effects of EZH2 and CCL2 on anti-inflammatory cytokines in HDPCs by qPCR (Figure 5). The results showed that expression of anti-inflammatory factors IL-4 and TGF-*β* decreased when the cells were stimulated with EZH2 complex for 24 hours (Figures 5(a) and 5(b)). But the expression of IL-10 increased (Figure 5(c)). We found the similar changes when the cells were stimulated with CCL2 complex and EZH2 plus CCL2 complex. In order to identify whether CCL2 inhibition could reverse the expression changes of anti-inflammatory factors induced by EZH2, we used EZH2 plus anti-CCL2 to treat HDPCs for 24 hours. EZH2 mediated decreasing of anti-inflammatory factors IL-4 and TGF-*β* could be upregulated by CCL2 inhibition (Figures 5(a) and 5(b)). The expression of IL-10 decreased in group of EZH2 plus anti-CCL2 compared with group of EZH2 (Figure 5(c)).

## 4. Discussion

Previous studies have confirmed that EZH2 can promote the progress of dental pulp inflammation by regulating the expression of cytokines *in vitro*. EZH2 can directly bind to the promoter of CCL2 and affect the transcription of CCL2 and promoting the progress of dental pulp inflammation [3]. However, the regulatory mechanism of EZH2 and CCL2 in the process of pulp inflammation and immune response remains to be studied. This study further confirmed that EZH2 participates in the development of dental pulp inflammation through *in vivo* and *in vitro* experiments. The expression of TNF-*α* was significantly upregulated in LPS-induced inflammatory pulp of rats. We found that the expression of EZH2 decreased within 8 hours of LPS-induced pulp inflammation. But after 1 day of stimulation, the expression of EZH2 gradually increased in time-dependent manner. The reduced expression of EZH2 in the early period of dental pulp inflammation may be related to the mechanism of repair in the early stage of dental pulp inflammation. This is consistent with our previous research [6]. And it might indicate that EZH2 might have important roles in regulating pulpitis. In the progress of pulpitis, there are lots of immune cells have been activated, such as macrophages and dendritic cells (DCs). Macrophages are one of the main cells involved in dental pulp inflammation. It plays an immunomodulatory role in dental pulp tissue due to strong phagocytic ability. A large number of macrophages are mainly distributed in the central region of dental pulp [19, 23]. In our study, a large number of CD68-positive cells infiltrated in the inflammatory pulp. Macrophages play a key role in maintaining tissue homeostasis and regulating immunity. It could be migrated to the injured sites of dental pulp by CCL2 chemotaxis [24]. In our previous study, we found that expression of CCL2 has been induced significantly by EZH2 in HDPCs [3]. So, we hypothesize that EZH2 might modulate the migration of macrophages in dental pulp inflammation. According to the results of immunofluorescence staining, we speculated that EZH2 might be able to regulate macrophages in dental pulp. And we found that EZH2 may promote the chemotaxis of macrophages by regulating CCL2. Therefore, it can be speculated that EZH2 in dental pulp can regulate the chemotaxis of macrophages via CCL2.

Studies have confirmed that dental pulp cells could participate in the immune response and regulate the inflammatory process of dental pulp [25]. Dental pulp cells also have certain immunomodulatory capabilities and can secrete inflammatory factors and anti-inflammatory factors [26]. Hui et al. confirmed that the stimulation of EZH2 complex could upregulate the expression of inflammatory factors of HDPCs, and the inhibitor of EZH2 could inhibit the inflammatory process of dental pulp [3]. However, the effect of EZH2 on the anti-inflammatory factors in dental pulp needs further research. In our study, the expression of anti-inflammatory factors IL-4 and TGF-*β* decreased when the HDPCs were stimulated with EZH2 and CCL2 complex compared with control, while the expression of IL-4 and TGF-*β* has been increased when the HDPCs were stimulated with EZH2 plus anti-CCL2, compared with EZH2 or CCL2 stimulation group. We speculated that EZH2 could promote pulp inflammation by inhibiting expression of anti-inflammatory factors. CCL2 inhibition can alleviate the reduced expression of anti-inflammatory factors affected by EZH2. However, the expression of anti-inflammatory factor IL-10 was contrary to IL-4 and TGF-*β*. It is reasonable to envisage that organism systems exist to modulate the response to microbial antigens by secreting immunoregulatory cytokines in order to prevent excessive inflammation. One of them might involve IL-10. Indeed, IL-10 is an immunosuppressive cytokine synthesized by many cell types, which decreases the production of several proinflammatory cytokines including IL-6 and CXCL8, thereby suppressing inflammation-associated immune responses and limiting damage to the host [27]. IL-10 could promote the differentiation of regulatory T cells which control excessive immune responses and then produce higher expression of IL-10. This might provide a positive regulatory loop for IL-10 induction [28]. We speculated that the increased expression of the anti-inflammatory factor IL-10 may be due to the existence of an immune response regulatory system which could prevent excessive inflammation. In recent years, many studies have found that EZH2 can play important roles in various inflammatory diseases such as lupus-like diseases, dental pulp inflammation, and neuropathic pain by promoting the expression of macrophage chemokine CCL2 [6, 29]. This is consistent with the results of our study.

However, there are also reports that intestinal mucosal epithelial EZH2 has a strong protective effect on experimentally induced inflammatory bowel disease [8]. This is diametrically opposed to the results of this study. It may be due to the different effects of EZH2 on different cells. Lim et al. found that EZH2 can regulate the adhesion and chemotaxis of inflammatory cells through methylating the protein talin directly [30]. Therefore, the mechanism of EZH2 promoting macrophage chemotaxis in pulpitis needs further exploration.

## 5. Conclusion

EZH2 might activate the macrophage chemotaxis and affect the transcription of anti-inflammatory factors via regulating the expression of CCL2 in the process of dental pulp inflammation.

## Data Availability

No data were used to support this study.

## Supplementary Materials

Supplementary Materials Supplementary Information: Supplementary Figure S1-5: primer sequences of genes detected by qPCR. Supplementary Figure S1-4: quantitative analyses of IHC/IF and transwell assay.
