# Supplementary material for: EZH2 Might Affect Macrophage Chemotaxis and Anti-Inflammatory Factors by Regulating CCL2 in Dental Pulp Inflammation
Source: Stem Cells Int. 2021 Dec 1;2021:3060480. doi: 10.1155/2021/3060480 (PMC8654562; doi:10.1155/2021/3060480)
Supplement: Supplementary Materials — Supplementary Information: Supplementary Figure S1-5: primer sequences of genes detected by qPCR. Supplementary Figure S1-4: quantitative analyses of IHC/IF and transwell assay. [file 3060480.f1.doc]

**Supplemental Materials:**


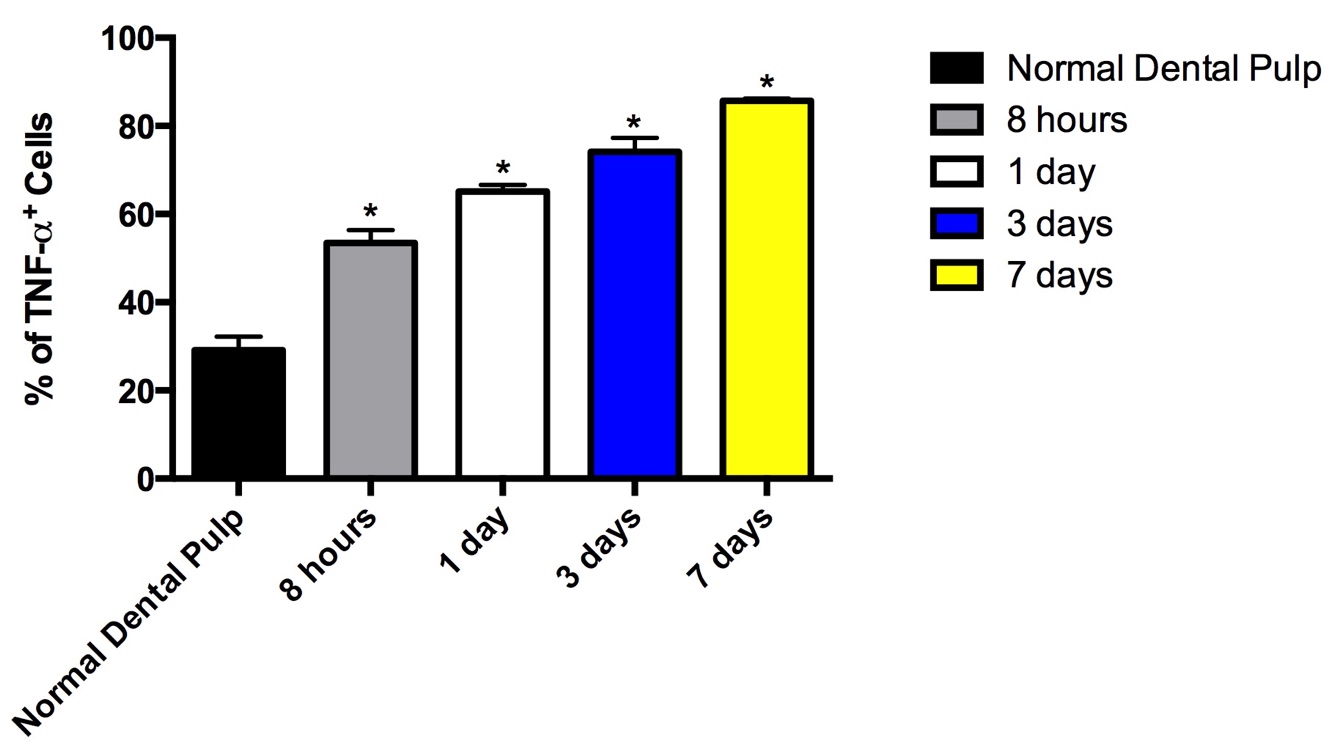


Respond to reviewer #1 Question 2: Fig S1: Quantitative analyses of TNF-α-positive cells. A significant increase in the numbers of TNF-α-positive cells was observed in LPS-treated rats compared to untreated rats (*P < 0.05; values are expressed as mean ± standard erros; n = 3 per group) Results of TNF-α staining revealed that dental pulp inflammation model has been established in LPS treated groups. (*P < 0.05, n=3 per group)


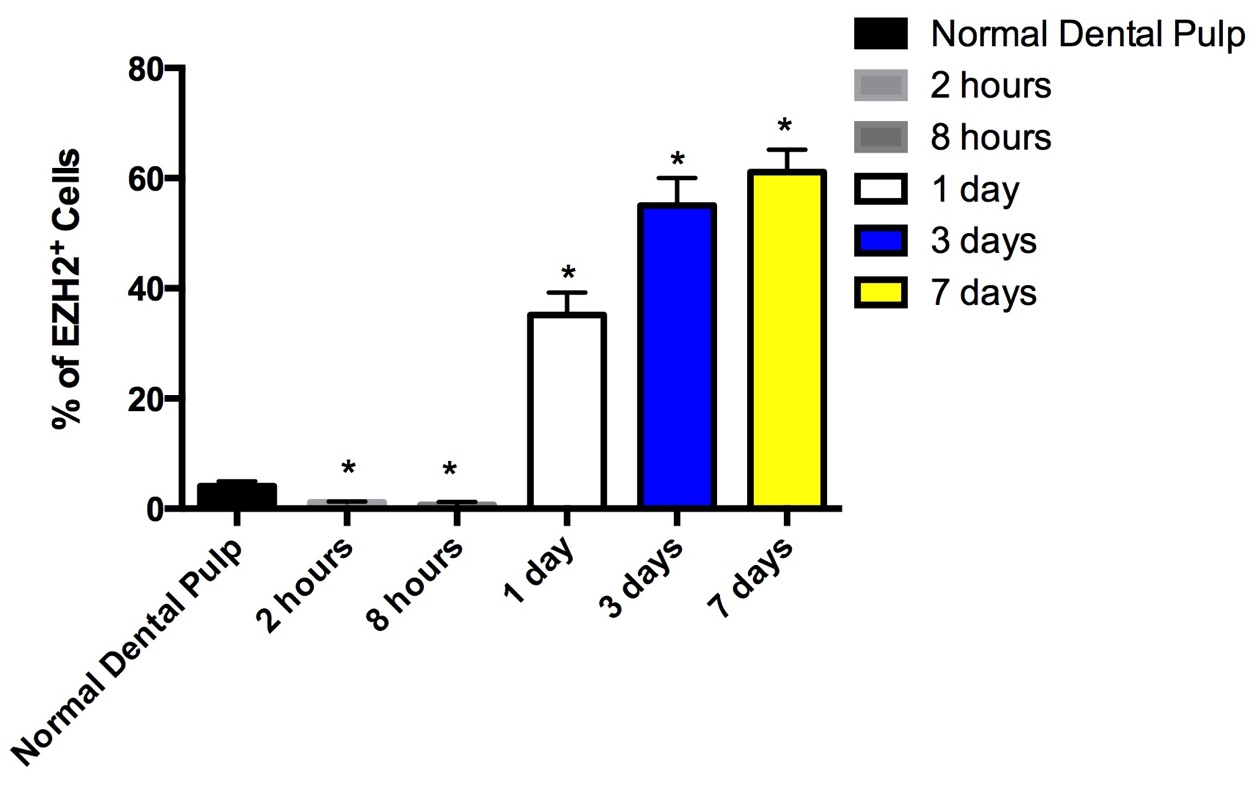


Respond to reviewer #1 Question 2: Fig S2: Quantitative analyses of EZH2-positive cells. Results of EZH2 staining revealed that the alteration of EZH2 expression in dental pulp inflammation of rats at different time points. (*P < 0.05, n=3 per group)

**A**  **B**


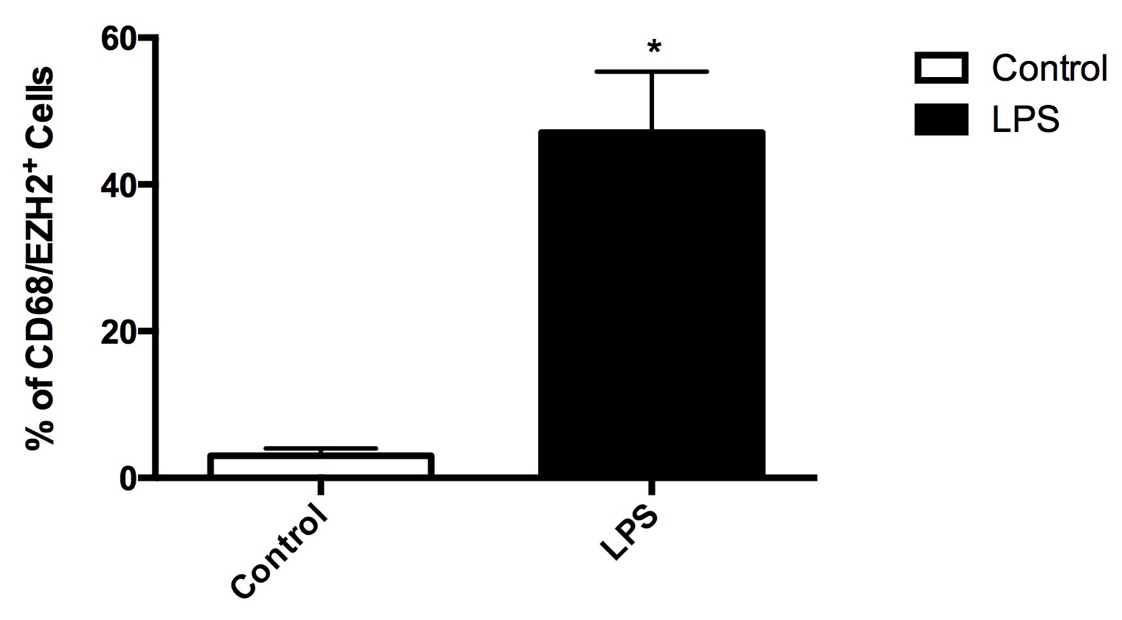

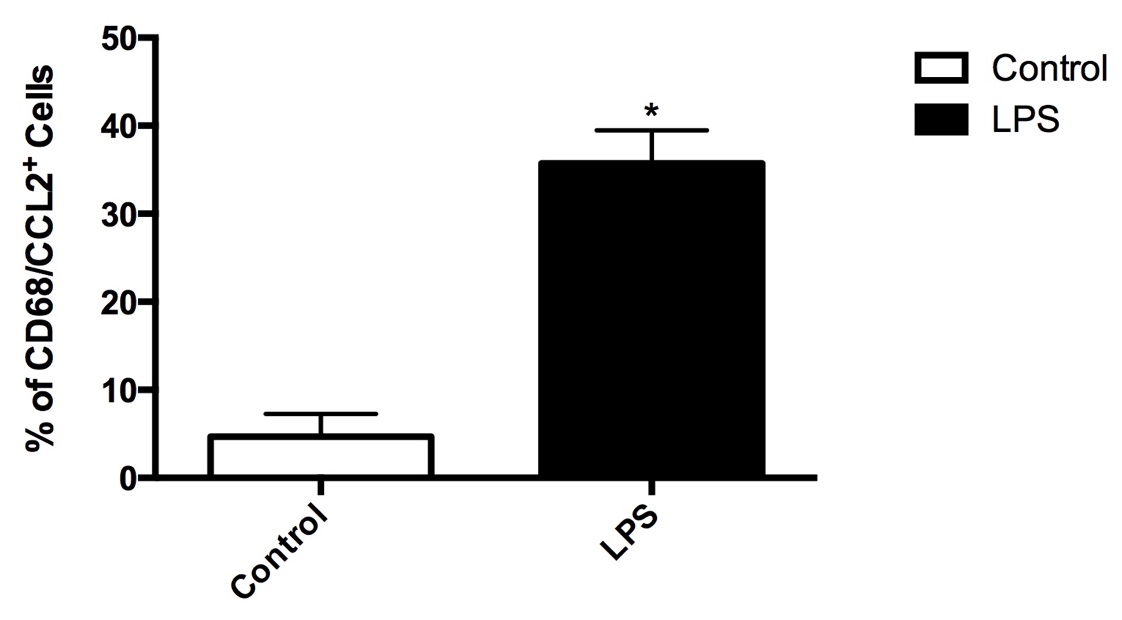


Respond to reviewer #1 Question 2: Fig S3: Quantitative analyses of CD68/EZH2-positive cells and CD68/CCL2-positive cells. Results of CD68/EZH2 (A) and CD68/CCL2 (B) staining revealed that the co-localization of CD68/EZH2 and CD68/CCL2 cells was significantly increased in LPS treated rats dental pulp compared with control. (*P < 0.05, n=3 per group)


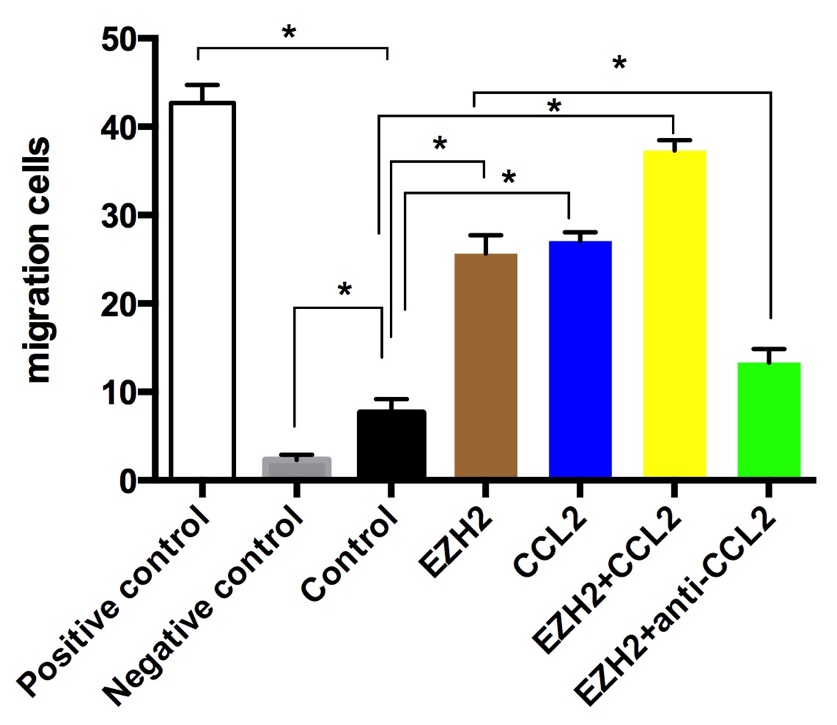


Respond to reviewer #1 Question 2: Fig S4: Quantitative analyses of migrated macrophages. macrophage migration has been induced by EZH2, CCL2 and EZH2 plus CCL2. The cell migration was suppressed by treatment with anti-CCL2 protein compared with EZH2 treated group.

Respond to reviewer #2 Question 5: Primer sequences of genes detected by qPCR are as follows: IL-4, F, 5,-AAAACTTTGAACAGCCTCACAG-3,, R, 5,- GGTTTCCTTCTCAGTTGTGTTC -3,, IL-10, F, 5,- GTTGTTAAAGGAGTCCTTGCTG,, R, 5,- TTCACAGGGAAGAAATCGATGA -3,, TGFβ, F, 5,- CTGTACATTGACTTCCGCAAG -3,, R, 5,- TGTCCAGGCTCCAAATGTAG -3,,

GAPDH, F, 5,-TCAACAGCGACACCCACTC -3,, R, 5,- GCTGTAGCCAAATTCGTTGTC -3,.


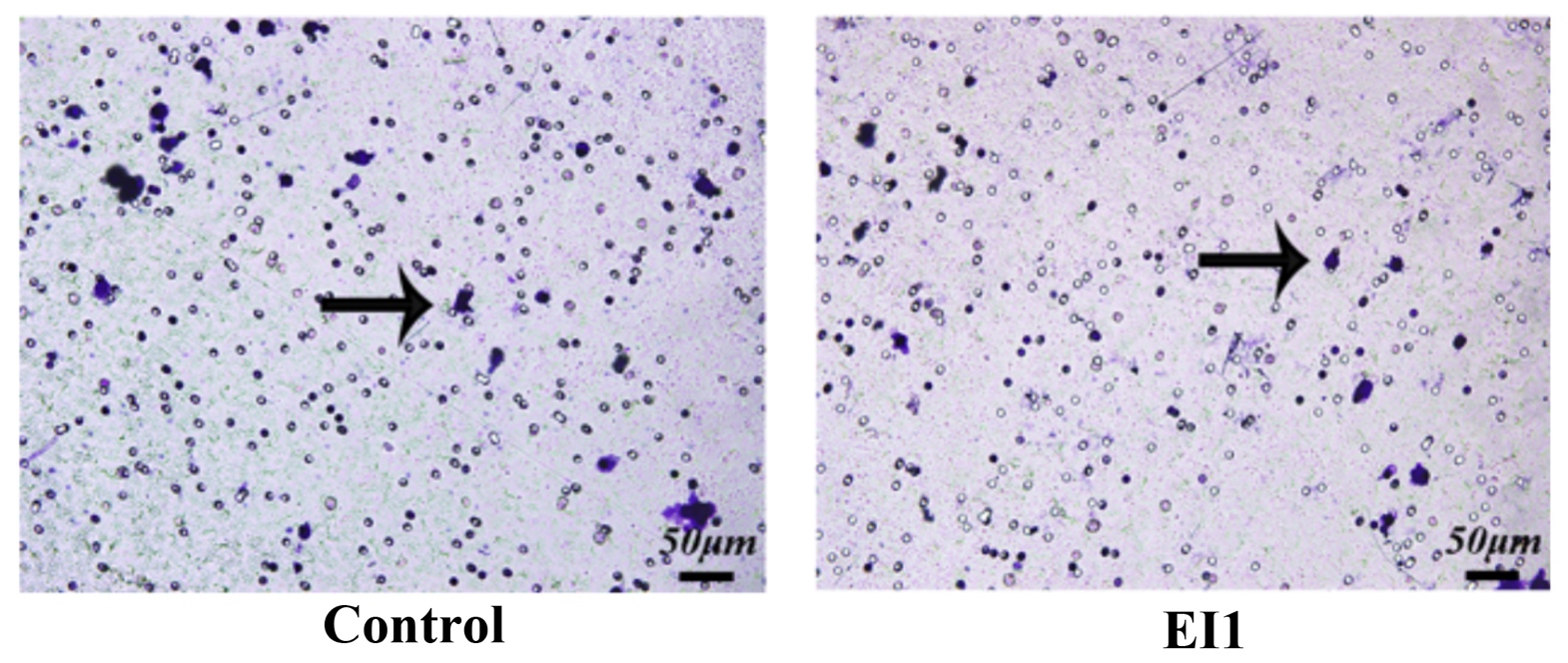


Respond to reviewer #2 Question 7: Fig S5: Chemotactic activity of EI1 treated HDPCs on macrophages. (scale bar: 50um)
